# Enhanced Performance of Perovskite Light-Emitting Diodes via Phenylmethylamine Passivation

**DOI:** 10.3390/mi13111857

**Published:** 2022-10-29

**Authors:** Shisong Yu, Kai Zhang, Xiangcheng Cai, Peng Tu, Yuanming Zhou, Fei Mei

**Affiliations:** 1 School of Electrical and Electronic Engineering, Hubei University of Technology, Wuhan 430068, China; 2 School of Science, Hubei University of Technology, Wuhan 430068, China

**Keywords:** perovskite, phenylmethylamine, chlorobenzene, light-emitting diode

## Abstract

Organic-inorganic perovskite materials are widely used in the preparation of light-emitting diodes due to their low raw material cost, solution preparation, high color purity, high fluorescence quantum yield, continuously tunable spectrum, and excellent charge transport properties. It has become a research hotspot in the field of optoelectronics today. At present, the nonradiative recombination and fluorescence quenching occurring at the interface between the device transport layer and the light-emitting layer are still important factors limiting the performance of perovskite light-emitting diodes (PeLEDs). In this work, based on CH_3_NH_3_PbBr_3_ perovskite, the effects of parameters such as precursor solution, anti-solvent chlorobenzene (CB), and small amine molecule phenylmethylamine (PMA) on the performance of perovskite films and devices were investigated. The research results show that adding an appropriate amount of PMA can reduce the grain size of perovskite, improve the coverage of the film, enhance the crystallinity of the film, and increase the fluorescence intensity of the perovskite film. When the PMA content is 0.050 vol.%, the maximum luminance of PeLEDs is 2098 cd/m^2^ and the maximum current efficiency is 1.592 cd/A, which is greatly improved by 30% and 64.8% compared with the reference device without PMA doping. These results suggest that an appropriate amount of PMA can effectively passivate the defects in perovskite films, and inhibit the non-radiative recombination caused by the traps, thereby improving the optoelectronic performance of the device.

## 1. Introduction

As an electro-optical conversion semiconductor device, light-emitting diodes are widely used in solid-state lighting and flat-panel displays [1,2,3,4,5,6]. In recent years, organic-inorganic hybrid perovskite materials have become a research hotspot in the field of light-emitting diodes due to their narrow half-peak width, tunable band gap, high fluorescence quantum yield, and bipolar charge transport [7,8,9,10,11,12,13]. Perovskite light-emitting diodes using halide perovskite materials as the light-emitting layer have attracted great attention from the academic community. In a relatively short period of time, the external quantum efficiencies of red and green perovskite light-emitting diodes (PeLEDs) jumped from 0.76% [14] and 0.1% [15] to 25.8% [16] and 28.1% [17], respectively.

The improvement of PeLED device performance is highly dependent on the surface morphology and crystal quality of the perovskite film, mainly by reducing the non-radiative recombination defects in the film and improving the crystallinity of the film, thereby improving the radiative recombination efficiency of carriers [18,19]. Studies have shown that small organic molecules can be introduced into the perovskite precursor solution to passivate defects or assist perovskite crystallization, thereby regulating the crystallization kinetics and reducing the formation of defects [20]. Yang et al. [21] inserted the small organic molecule trioctylphosphine oxide (TOPO) between the quasi-two-dimensional perovskite layer and 2,2′,2′-(1,3,5-benzinetriyl)-tris(1-phenyl-1-H-benzimidazole) (TPBi), and the ligands in TOPO reacted with the incomplete inorganic octahedra in the perovskite to form chemical bonds. The perovskite surface was passivated, the fluorescence quantum yield (PLQY) of the perovskite film after passivation increased from 57.3% to 73.8%, and the fluorescence lifetime increases from 0.17 μs to 0.36 μs, indicating that the nonradiative recombination defects at the interface are effectively suppressed. Ma et al. [22] reported a fluorinated triphenylphosphine oxide to perform surface passivation and well-width modulation of low-dimensional perovskite materials, and obtain efficient reduced-dimensional perovskite LEDs with more monodispersed quantum well thickness distribution. The as-prepared green devices exhibit a high external quantum efficiency of 25.6% and an operating lifetime of 2 h at an initial brightness of 7200 cd/m^2^. In addition to introducing small organic molecules for defect passivation, controlling the crystallization kinetics of perovskite films can also effectively suppress the formation of defects in films, in which the most commonly used method is anti-solvent treatment [23]. Kong et al. [24] developed an anti-solvent method with an ultra-wide time processing window to prepare perovskite thin films by introducing anti-solvent into perovskite for one-step spin coating, and highly crystalline and dense perovskite films were successfully prepared.

In this paper, the perovskite precursor solutions were prepared by using chlorobenzene (CB) as the anti-solvent and small amine molecule phenylmethylamine (PMA) as the passivation agent respectively. The effects of PMA on the crystalline quality, coverage, and surface defects of perovskite films were systematically studied, as well as the performance improvement of PeLED devices.

## 2. Materials and Methods

### 2.1. Materials

**Figure 1:**
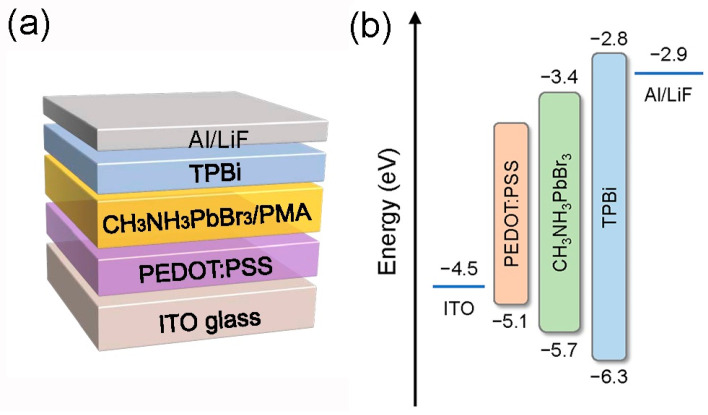
The schematic structure (**a**) and energy level diagram (**b**) of the PeLEDs.

The device structure used in this work is ITO/PEDOT:PSS/CH_3_NH_3_PbBr_3_/TPBi/LiF/Al, as shown in Figure 1. Poly(3,4-ethylenedioxythiophene):poly(styrene sulfuric acid) (PEDOT:PSS, Clevios P AI4083), Methylammonium bromine (CH_3_NH_3_Br, 99.5%), Lead Bromide (PbBr_2_, 99.999%), TPBi were purchased from Xi’an Polymer Light Technology corp. N,N-Dimethylformamide (DMF, 99.9%) and phenylmethylamine (PMA, 99%) were purchased from Sigma-Aldrich Co., Ltd. (St. Louis, MO, USA). Dimethyl sulfoxide (DMSO) and chlorobenzene (CB) were purchased from Shanghai Aladdin Bio-Chem Technology Co., Ltd. (Shanghai, China). Aluminum slug (Al, 99.999%) was purchased from Alfa Aesar (Haverhill, MA, USA). The substrate is indium tin oxide (ITO), which has the characteristics of transparency and strong conductivity, and is widely used in light-emitting devices. The square resistance of ITO is ~15 Ω/m^2^, and the effective light-emitting area is 0.1 cm^2^. Firstly, the ITO substrate was ultrasonically cleaned with isopropanol for 30 min and then washed with absolute ethanol and ultrapure water for 10 min each. Contamination on the surface of the substrate is cleaned to prevent it from affecting the subsequent spin-coating process. Subsequently, the moisture on the surface of the substrate was blown off with a nitrogen gun, and then transferred to an oxygen plasma chamber for cleaning for 20 min. The precursor solution was then prepared by dissolving CH_3_NH_3_Br and PbBr_2_ in DMF/DMSO solvent at a molar ratio of 1.05:1. In order to obtain perovskite film with PMA treatment, PMA-chlorobenzene mixed antisolvent solution were prepared by dissolving an appropriate amount of PMA solution (0, 0.025 vol.%, 0.050 vol.%, 0.075 vol.%) in the anti-solvent chlorobenzene (CB), and then stirred at 60 °C for 12 h in the dark environment [25,26,27].

### 2.2. Device Fabrication

Firstly, the hole transport layer and the light-emitting layer were prepared, and PEDOT:PSS was filtered and ultrasonically stirred to obtain a uniformly dispersed solution. A total of 50 μL of PEDOT:PSS solution was added dropwise onto ITO, spin-coated at 5000 rpm for 40 s, and annealed at 140 °C for 10 min to obtain a uniform and dense PEDOT:PSS film. Then the perovskite precursor solution (CH_3_NH_3_PbBr_3_) was added dropwise to the hole transport layer for spin coating at 7000 rpm, and 100 μL of PMA-chlorobenzene mixed antisolvent solution was added dropwise at 30 s after the beginning of spin-coating to obtain the CH_3_NH_3_PbBr_3_ film [28,29]. The samples spin-coated with the hole layer (PEDOT:PSS) and the light-emitting layer (CH_3_NH_3_PbBr_3_) were transferred to an evaporation chamber with the vacuum degree of 10^−7^ Torr, and then the electron transport layer (TPBi), electron injection layer (LiF) and electrode (Al) were sequentially evaporated. Finally, the devices were encapsulated with a glass cover slip and AB glue.

### 2.3. Device Characterization

A stylus profiler (Alpha-Step D-600, KLA Corporation, Milpitas, CA, USA) was used to measure the thickness of functional layers such as pure PEDOT:PSS films and perovskite films. A luminescence spectrometer (HITACHI F-4600, Hitachi Limited, Hitachi, Japan) was used to measure the photoluminescence (PL) spectra of the films (emission wavelength at 315 nm). Scanning electron Microscopy (SEM, FEI Sirion FEG, FEI Corporation, Eindhoven, The Netherlands) and X-ray diffractometer (XRD, Malvern Panalytical Empyrean Nano, PANalytical B. V., Almelo, The Netherlands) were used to measure the surface morphology and crystal structure of the perovskite films respectively. A digital source meter (Keithley 2400, Tektronix, Inc., Beaverton, OR, USA), a digital multimeter (Keithley 2000, Tektronix, Inc., Beaverton, OR, USA), and a silicon photodetector were connected to form an L-I-V test system, which is used to detect the photoelectric characteristics of PeLEDs.

## 3. Results and Discussion

The preparation of perovskite thin films by one-step spin coating has become an effective method because of its simple and fast operation method. However, this method is difficult to control the nucleation and growth rate of the film, and it is easy to introduce defects in the film, resulting in non-radiative recombination loss of the device [30]. In this paper, the ratio of nucleation and growth rate of perovskite films is regulated by introducing an anti-solvent during spin coating. In order to make the anti-solvent better improve the quality of the perovskite film, it is necessary to systematically study the ratio of the precursor solution and select the optimal ratio.

**Figure 2:**
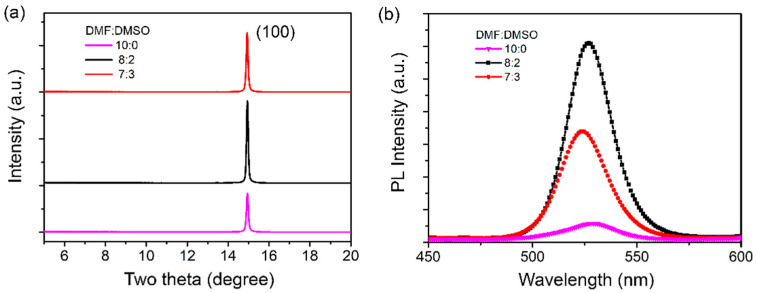
XRD patterns (**a**) and PL spectra (**b**) of the CH_3_NH_3_PbBr_3_ films with different ratios of precursors.

Figure 2a shows the X-ray diffraction (XRD) patterns of perovskite thin films prepared with different DMF:DMSO ratios of solvent in the precursor solution. All the samples have diffraction peaks around 2θ = 14.9°, which correspond to the typical (100) crystal planes. The XRD patterns of the perovskite films corresponding to different DMF:DMSO ratios have similar peak positions, indicating that the incorporation of DMSO does not change the crystal structure of the perovskite. Moreover, it can be clearly seen that when the ratio of DMF:DMSO is 8:2, the intensity of diffraction peak is enhanced, indicating that the crystallinity of perovskite film prepared by this precursor solution was improved. Figure 2b shows the PL spectra of perovskite films prepared with different DMF:DMSO ratios. The conclusion is basically consistent with the XRD analysis results. When the ratio of DMF:DMSO is 8:2, the optimal optical properties of perovskite film were obtained.

**Figure 3:**
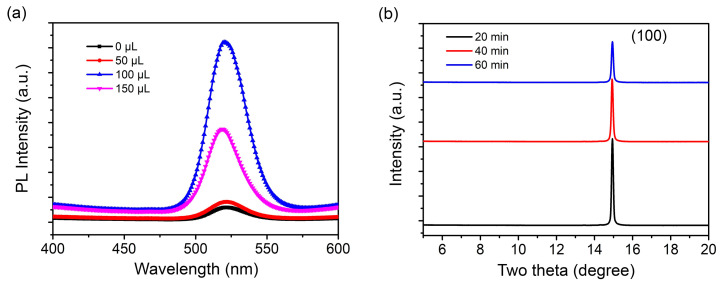
The PL spectra (**a**) of CH_3_NH_3_PbBr_3_ films with different volumes of anti-solvent CB and XRD patterns (**b**) of CH_3_NH_3_PbBr_3_ films with different annealing times.

The antisolvent can affect the coverage and crystallization kinetics of perovskite crystals, thereby affecting the perovskite grain morphology. To further determine the experimental parameters of the anti-solvent CB, the effects of the added amount of the anti-solvent CB and the annealing temperature on the perovskite films were investigated. Figure 3a shows the PL characteristics of CH_3_NH_3_PbBr_3_ films with different volumes of anti-solvent CB. The PL intensity was significantly enhanced with the addition of CB from 0 μL to 100 μL, and the full width at half maximum is also slightly reduced from 33.2 nm to 31.5 nm. It shows that the addition of CB can passivate the defects in the material and improve radiation recombination. When the addition amount of CB was further increased to 150 μL, the PL intensity began to decrease, indicating that the excess anti-solvent may induce perovskite precipitation, which results in the deterioration of the crystal quality of the film. [31,32,33].

At the same time, the thermal annealing process plays an important role in the preparation of uniform perovskite films. The perovskite films prepared with the above parameters were annealed at 80 °C for different time periods. Figure 3b shows the XRD spectra of perovskite films with different annealing times. The results show that all samples have diffraction peaks around 2θ = 14.9°, which correspond to the typical (100) crystal plane. According to the XRD patterns for different annealing times, the better crystallinity of perovskite can be obtained by annealing for 20 min. On increasing the annealing time, the diffraction peaks of the perovskite films gradually decrease, indicating that too high annealing temperature will lead to discontinuous morphology and cracks in the perovskite films, thus affecting the crystal quality of the perovskite films [34,35].

**Figure 4:**
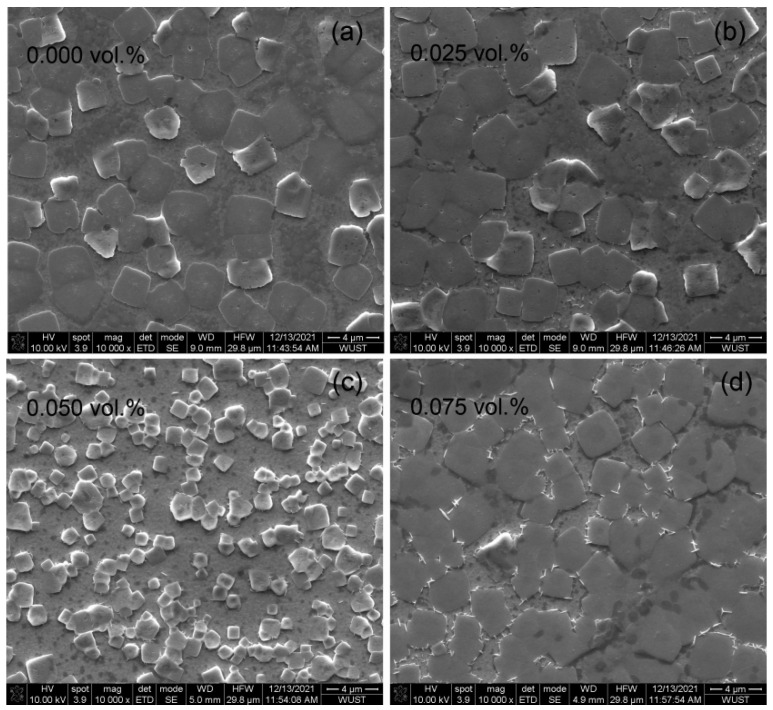
The SEM images of CH_3_NH_3_PbBr_3_ films (**a**) without PMA doping, (**b**) with 0.025 vol.% PMA, (**c**) with 0.050 vol.% PMA, (**d**) with 0.075 vol.% PMA.

The diversity and variable molecular structure of small amine molecules can enhance the optoelectronic properties of perovskite devices through strong chemical interactions with perovskites. The commonly used method is mainly to introduce small amine molecules into the perovskite precursor solution to passivate defects or assist perovskite crystallization, thereby regulating the crystallization kinetics and reducing the formation of defects [34,35,36,37,38,39]. On the basis of the previous studies, the effect of small amine molecule phenylmethylamine (PMA) on the surface morphology, crystal structure, and optical properties of perovskite films was explored. The surface morphology of the perovskite films treated with different concentrations of PMA was observed by SEM. As shown in Figure 4, the perovskite grains prepared without PMA were large in size, reaching 600–900 nm. When PMA was added to 0.050 vol.%, the particle size decreased gradually, suggesting the better spatial confinement of excitons. However, when the PMA concentration further increase to 0.075 vol.%, the perovskite thin films showed some crystal shapes such as burrs or rods, which were due to the formation of a two-dimensional (2D) layered perovskite structure [35].

**Figure 5:**
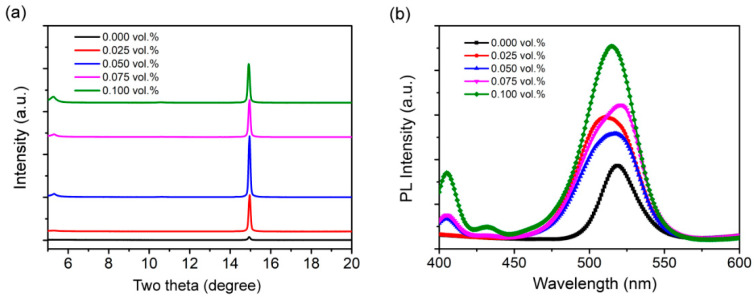
The XRD patterns (**a**) and PL spectra (**b**) of CH_3_NH_3_PbBr_3_ films with different concentrations of PMA.

In order to further explore the effect of PMA on perovskite films, XRD analysis was performed on the perovskite films prepared with different concentrations of PMA, as shown in Figure 5a. When the PMA concentration is less than 0.050 vol.%, the XRD pattern only exhibits a diffraction peak position of 14.9°, which corresponds to a typical three-dimensional perovskite structure. When the PMA concentration is greater than or equal to 0.050 vol.%, the film shows an additional XRD diffraction peak position at 5.28°, and with the increase of PMA concentration, the XRD peak intensity at 5.28° increases, while the intensity of the 14.9° diffraction peak decreases, indicating the transition from the three-dimensional (3D) to the 2D layered structure. Consistent with the structure of the XRD analysis, the PL results (Figure 5b) show that the perovskite films prepared with a high concentration of PMA have a large band gap due to the formation of a 2D layered perovskite structure. In addition to the main peak at 523 nm, an additional PL peak was found at 404 nm. From the above results, it can be seen that a high concentration of PMA will lead to the formation of a 2D layered structure in 3D perovskite [35,36,37]. On the one hand, the amine passivation strategy can reduce the trap-state density and suppress the non-radiative recombination, and a high recombination probability is achieved by confining the charges within the small grains, which can enhance the efficiency of light-emitting diodes [37]. On the other hand, the bulky amine ligand in the 2D layered structure may interrupt charge transport in the perovskite layers, which can reduce the efficiency of light-emitting diodes [34,37]. Therefore, the selection of suitable content of small amine is particularly important for the preparation of perovskite thin films.

**Figure 6:**
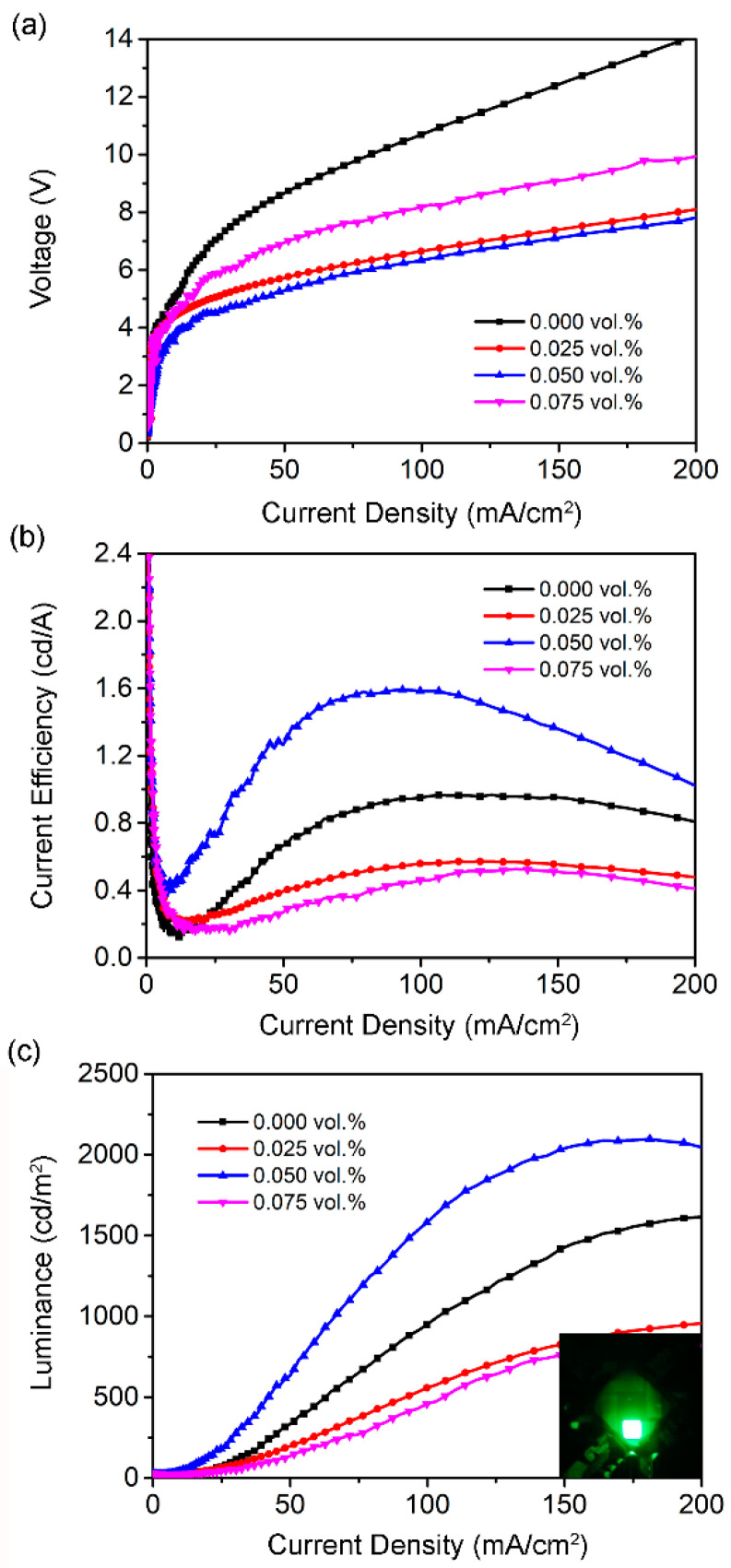
(**a**) the current density-voltage (J-V), (**b**) the current efficiency-current density (CE-J), and (**c**) the luminance-current density (L-J) curves for the CH_3_NH_3_PbBr_3_ PeLEDs with different concentration of PMA.

In order to study the effect of PMA treatment on the photoelectric properties of PeLED devices, perovskite light-emitting devices with the structure ITO/PEDOT:PSS/CH_3_NH_3_PbBr_3_/PMA/TPBi (40 nm)/LiF (1 nm)/Al (110 nm) was prepared. The structure diagram and energy band diagram are shown in Figure 1. Figure 6a–c are the voltage-current density (V-J), current efficiency-current density (CE-J), and luminance-current density (L-J) curves of perovskite light-emitting devices prepared with different concentrations of PMA, respectively. The detailed device parameters of PeLEDs with a different doping concentration of PMA can be found in Table 1. When the PMA concentration is 0.050 vol.%, the device exhibits the best optoelectronic properties. The maximum brightness is 2098 cd/m^2^ and the maximum CE is 1.592 cd/A, which is 30% and 64.8% higher than that of the control device without PMA doping, respectively. As shown in Figure 6a, the turn-on voltage with PMA doping is reduced compared with the control device. These results demonstrate that passivation via PMA leads to reduced trap density in perovskite films, suppresses trap-induced non-radiative decay by limiting the number of defects, and achieves high recombination probability by confining charges within small grains, thereby achieving high recombination probability. The luminous efficiency and charge transport properties of perovskite films were improved [36]. However, the devices showed lower device luminance with further increasing PMA concentration to 0.075 vol.%, suggesting that the bulky amine ligands in the 2D perovskite structure interrupt the charge transport in the perovskite layer, leading to the reduced device performance.

## 4. Conclusions

In this paper, the small amine molecule PMA was introduced into PeLED devices to study the effects of experimental parameters on the optoelectronic properties of perovskite films and devices. When a suitable amount of PMA is introduced into the perovskite film, the reduced grain size, the enhanced density, the increased coverage, and the obtained for the perovskite films, which greatly improve the PL intensity of the perovskite film. When the PMA content is 0.05 vol.%, the maximum brightness of the perovskite light-emitting device is 2098 cd/m^2^, and the maximum CE is 1.592 cd/A, which is greatly improved compared with the control device. This is because an appropriate amount of PMA can passivate the defects of the perovskite film, suppress the trap-assisted nonradiative decay, and make the perovskite film have a high radiative recombination efficiency. This study will provide a simple and effective method to improve the luminous efficiency of perovskite light-emitting diodes.

## Figures and Tables

**Table 1:** Summary of the device parameters of PeLEDs with a different doping concentration of PMA.

PMA Doping Content	w/o	0.025%	0.050%	0.075%
L_max_ (cd/m^2^)	1615	956	2098	827
CE_max_ (cd/A)	0.966	0.572	1.592	0.527

## Data Availability

Not applicable.
